# CYP1A1, mEH, and GSTM1 Polymophisms and Risk of Oral and Pharyngeal Cancer: A Spanish Case-Control Study

**DOI:** 10.1155/2008/741310

**Published:** 2008-09-22

**Authors:** L. Varela-Lema, A. Ruano-Ravina, M. A. Juiz Crespo, K. T. Kelsey, L. Loidi, J. M. Barros-Dios

**Affiliations:** ^1^Department of Preventive Medicine and Public Health, University of Santiago de Compostela, 15782 Santiago de Compostela, Galicia, Spain; ^2^Galician Agency for Health Technology Assessment, Galician Health Service, 15782 Santiago de Compostela, Galicia, Spain; ^3^Biomedical Research Centre Network for Epidemiology and Public Health (CIBERESP), 08003 Barcelona, Spain; ^4^Centro de Saúde Porto do Son, 15970 A Coruña, Spain; ^5^Department of Genetics and Complex Diseases, Harvard School of Public Health, MA 02115, USA; ^6^Unit of Molecular Medicine, Santiago de Compostela University Hospital, Galician Health Service, 15706 Santiago de Compostela, Galicia, Spain

## Abstract

*Background*. Genetic polymorphisms of drug metabolizing enzymes involved in the detoxification pathways of carcinogenic substances may influence cancer risk. *Methods*. Case-control study that investigates the relationship between CYP1A1 Ile/Val, exon 4 mEH, and GSTM1 null genetic polymorphism and the risk of oral and pharyngeal cancer examining the interaction between these genes, tobacco, and alcohol. 92 incident cases and 130 consecutive hospital-based controls have been included. *Results*. No significant associations were found for any of the genotypes assessed. The estimated risk was slightly elevated in subjects with the wild type of the mEH gene and the null GSTM1 genotype. For exon 4 mEH heterozygous polymorphism, the risk was slightly lower for heavy smokers than for light smokers. The inverse association was observed for the GSTM1 null genotype. *Conclusions*. The results suggest that exon 4 mEH and GSTM1 null polymorphisms might influence oral and pharyngeal cancer.

## 1. Introduction

Oral and pharyngeal cancers represent an important problem worldwide. The incidence and prevalence rates for these tumors are double in men than in women. Cancers of the oral cavity rank as the eighth most common cancer among men, being responsible for 3% of the cancers diagnosed in this gender [1]. Mortality rates are substantially lower than incidence rates. According to the World Health Organization (WHO) data, the standardized mortality rate for 2002 was 2.2 deaths per 100 000 population.

Tobacco and alcohol are the main risk factors for oral and pharyngeal cancers. In USA and Europe, they are responsible for 75–80% of these tumors [2, 3]. Epidemiologic studies performed in all continents have found an increased risk in smokers, which seems to rise with daily consumption and duration [4–7]. There is also sufficient evidence to conclude that excessive consumption of alcoholic beverages is associated with oral and pharyngeal cancers, causing in some cases risks higher than those found for smokers [8–10].

The reason why some individuals develop cancer and others do not can be attributed, at least partly, to variations in genetic polymorphisms responsible for metabolizing carcinogenic substances found in tobacco and alcohol. Although many genes have been associated with metabolism of these compounds, some with the highest rational of those being involved are CYP1A1, mEH, and GSTM1.

The CYP1A1 gene belongs to the CYP1 subfamily and encodes for the enzyme aryl hydrocarbon hydrolase, which is involved in the activation of many polycyclic aromatic hydrocarbons and aromatic amines [11] and is present in oral tissue [12]. This enzyme is implicated in the metabolism of benzo[a]pyrene, a potent tobacco carcinogen. Various studies have shown that CYP1A1 catalyzes the initial conversion of benzo[a]pyrene to 7,8 dihydrodiol-9,10-oxide [13, 14]. Two CYP1A1 polymorphisms have been related to different tumors, including head and neck cancers. One of these is a single-base substitution of adenine to guanine at position 2455 in the heme-binding region of exon 7, which induces an amino acid change in isoleucine to valine at codon 462, known as the Ile/Val or exon 7 polymorphism (Ile 462 Val) or CYP1A1*2C. This mutation has also been referred to as mutation m2. The Ile/Ile genotype corresponds to the wild type, and Ile-Val and Val-Val to the heterozygous and homozygous genotypes for the mutant allele, respectively [15]. This mutation is rare in Caucasians, and is in complete linkage disequilibrium with the CYP1A1 MspI mutation (CYP1A1*2B) [16].

The microsomal form of epoxide hydrolase is primarily associated with the metabolism of exogenous xenobiotic compounds. Its interest in oral and pharyngeal cancers comes from the fact that it has been detected in all tissues including the aerodigestive tract and catalyzes the hydrolosis of arene, alkene, and aliphatic epoxides from polycyclic aromatic hydrocarbons and aromatic amines [17]. Enzymatically, mEH catalyzes the hydrolysis of epoxides to trans-dihydrodiols [18, 19]. Two polymorphisms in the mEH gene have been reported [20, 21]. The first polymorphism is produced as a consequence of a substitution of C → T within exon 3 of the gene and results in a substitution of His to Tyr in amino acid position 113. This polymorphism is known as the “slow allele” since in vitro studies show a 40–60% decrease in enzyme activity in comparison to the wild type. This allele is also known as HYL*2. In the second polymorphism, G substitutes A in exon 4, leading to an emplacement of histidine for arginine in the amino acid position 139 (139 Arg → His). This polymorphism is known as the fast allele HYL*3 since it produces a 25% increase in enzyme activity in vitro. Tyr is the predominant amino acid at the 113 position in Caucasian populations, and His is the most predominant at position 139.

The glutathione S transferase (GST) comprises a family of phase II detoxifying enzymes that catalyze a great number of detoxification reactions that take place between the cytosolic glutathione and compounds containing an electrofilic centre [22]. The GST substrates include acetaldehyde and several polycyclic aromatic hydrocarbons found in tobacco smoke. The GSTM1 is involved in the detoxification of benzo[a]pyrene-7,8-diol-9,10-oxide [23]. Metabolism of this carcinogen involves a balance between the activation steps mediated by the epoxide hydrolase and the cytochrome system and the detoxification steps, involving GSTM1, that inhibit the activity of the DNA binding intermediates and catalyze the conversion of the reactive electrophiles to inactive, water-soluble conjugates that can be easily removed [13]. Even though frequencies of GSTM1 null genotypes vary among different ethnic groups, in white Caucasians, it is deleted in approximately 50% of the population [24–33].

In the present case-control study, we aimed to examine the relationship between the CYP1A1 Ile/Val, exon 4 mEH (139 Arg → His), and GSTM1 null genetic polymorphism and the risk of oral and pharyngeal cancers, investigating also the association with smoking, drinking, and the gene-gene interactions.

## 2. Patients and Methods

### 2.1. Design, Subjects, and Settings

The present hospital-based case-control study was conducted at the Santiago de Compostela University Hospital Complex (Galicia, NW Spain) between January 1996 and January 2000. Data was collected on a total of 92 incident Caucasian male cases with histopathologically confirmed diagnosis of primary oral or pharyngeal cancers. The study was restricted to newly diagnosed patients over 20 years of age without a prior history of cancer. For study purposes, tumors of the lip were excluded.

A total of 130 consecutive controls were included from patients attending the Hospital Complex Preoperative Unit for nonsmoking- and nonalcohol-related trivial surgery. The inclusion criterion for controls was absence of prior history of cancer. Subjects under 20 years of age were excluded. The types of surgical procedures controls were scheduled to undergo mainly comprised inguinal hernias, cataracts, and orthopedic surgery. Informed consent was obtained from all study subjects prior to the interview and the extraction of total blood. Only 1 case and 6 controls refused to participate in the study. The study protocol was approved by the Galician Ethical Research Committee.

### 2.2. Information Retrieval

All study subjects were interviewed by a person purpose-trained to administer a structured questionnaire addressing various aspects of lifestyle, with special emphasis on smoking habit, alcohol consumption, occupation, and other activities related to the development of oral and pharyngeal cancers. For cases, the interview was done as soon as possible after cancer was detected and always within 15 days from diagnosis. The questionnaire used was previously applied to a group of individuals fulfilling the characteristics of the subjects going to be included and all questions with difficult interpretation were duly amended.

### 2.3. Laboratory Methods

After cases and controls were identified, whole blood samples of 6 mL were collected from each subject in heparin-containing tubes. The samples were stored at 4°C and centrifuged at 2800 rpm for at least 10 minutes within the next 24 hours. The three independent fractions were isolated and stored at −84°C until analysis. DNA was extracted from the buffy coat of middle layer containing monocytes. The cells were washed with TE (10x) and centrifuged at 3000 rpm during 10 minutes several times until they were cleaned. The pellet was then treated with lysis buffer and proteinase K in 1% SDS previous to the extraction of DNA with phenol-chloroform and ethanol precipitation similarly as previously described [34]. The DNA was precipitated in the presence of high concentrations of ammonium acetate (to further purify the DNA) and resuspended in TE to approximately 300 *μ*g/mL. The genotyping assays were performed at the Molecular Medicine Unit of the Santiago de Compostela University Hospital Complex.

Genotyping for GSTM1 was carried out in the whole sample using a modified PCR method described previously [35]. Reactions were carried out in a final volume of 12.5 *μ*L containing 20 mM Tris-HCl (PH 8.4), 50 mM KCl, 3 mM MgCl_2_, 0.25 *μ*M of each GSTM1 primer, 0.25 *μ*M of each *β*-globin primer (internal control), 1 U Taq DNA polymerase (Promega), 2 *μ*M each dNTPs, and 600–900 ng of DNA. The GSTM1 primers were 5′-GAACTCCCTGAAAAGCTAAGC and 5′-GTTGGGCTCAAATATACGGTGG. A negative control was included in all batches. The PCR conditions were 94° for 3 minutes, followed by 35 cycles of 94°C for 30 seconds, 58°C for 30 seconds, 72°C for 45 seconds, and a final extension of 72°C for 7 minutes.

To analyse the exon 4 mEH polymorphism (EH^139 arg^), a 357-bp fragment containing the polymorphic site was amplified [36]. The PCR was performed in all subjects using the sense primer 5′-GGGGTGCCAGAGCCTGACCGT-3′ and the antisense primer 5′-AACACCGGGCCCACCCTTGGC-3′ (Sigma-Genosys). The PCR cycling conditions were 95°C for 2 minutes, followed by 35 cycles at 95°C for 30 seconds, 60°C for 30 seconds, 72°C for 30 seconds, with a final step at 72°C for 7 minutes. After PCR amplification, 10 *μ*L of the PCR product was digested overnight at 37°C with 10U of RsaI (Invitrogen). The wild-type genotype (AA) produced 295-bp and 62-bp bands, the heterozygous genotype (AG) yielded 295-bp, 174-bp, 121-bp, 62-bp; and the rare allele (GG) gave 174-bp, 121-bp, and 62-bp bands.

The Ile-Val polymorphism was analysed in only 158 of the 222 individuals included using an allele-specific oligonucleotide-PCR procedure previously described by Hayashi et al. [16]. In the same reaction mix, two primers with different terminal bases (1A1A or 1A1G), which contained the polymorphic site at the 3′end, were added (1A1G : 5′-GAACTGCCACTTCAGCTGTCT-3′ and 1A1A : 5′-AAGACCTCCCAGCGGGCAAT-3′) in conjunction with another strand of primer (1A1.1 : 5′-GAACTGCCACTTCAGCTGTCT-3′). Two amplification reactions were necessary for each one of the subjects analysed, one with the primers 1A1.1/1A1A which recognize the Ile462 allele and another with the primers 1A1.1/1A1G which recognize the Val462 allele. PCR was carried out at 30 cycles under the following conditions: 30 seconds at 95° for denaturing, 1 minute at 60°C for primer annealing, and 1 minute at 72°C for primer extension.

All the products were electrophoresed on a 2% agarose gel stained with ethidium bromide and visualized under UV light. To test for eventual contamination, negative controls were introduced in each run.

### 2.4. Statistical Analysis

Logistic regression was employed to analyse the effect of each of the genes studied. The wild type gene was considered as the reference category for each gene in each analysis. All the regressions were adjusted for age, tobacco consumption in pack-years, and alcohol consumption in grams/week. For analyzing the effect of the different polymorphisms across different categories of tobacco and alcohol consumption, the cutpoint was established on the median creating two categories, low and high consumption in order to achieve a higher statistical power. In all analyses, the dependent variable was the status of case or control and the risks were expressed as ORs with CI 95%. All the analyses were performed with SPSS 11.5 statistical package.

## 3. Results

The study population covered a total of 222 subjects, comprising 92 cases and 130 controls. A description of the sample characteristics is presented in Table 1. The mean age of the controls (59.4 years; CI 95% 57–61.8) was slightly higher than that of cases (55 years; CI 95% 52.7–57.3). The 57.6% of the tumors were located in the oral cavity (tongue 27, oral floor 7, palate 6, and other parts 12) and 96.7% were squamous cell carcinomas. Smoking and alcohol consumption were more frequent among cancer cases than controls. A percentage of 98% of the cases were smokers and 46.7% of these (*n* = 43) were considered as heavy drinkers (more than six glasses of wine/beers or two liquors a day), whilst in the control group 62% of the individuals smoked and 10.8% (*n* = 14) were heavy drinkers.

The frequency of GSTM1 null genotype was 51.1% among oral and pharyngeal cancer patients and 47.7% among controls. The exon 4 mEH (139 Arg → His) heterozygous polymorphism (His/Arg) was present in 31.5% of the control group and in 27.2% of the case group. Only 4 subjects (4.3%) in the case group and 1 subject in the control group (0.8%) presented homozygous mutations. There were no differences in the frequency of mutations for the CYP1A1 exon 7 polymorphism. The mutant alleles (Ile/Val, Val/Val) were observed in only 2 cases (3%) and 2 controls (2.2%).

Table 2 shows the odds ratio estimates for combined oral and pharyngeal cancers associated with the GSTM1, CYP1A1, and exon 4 mEH polymorphisms. To determine the effect of these polymorphisms on different cancer sites, oral cavity and pharyngeal tumors were evaluated separately. No statistically significant effect was observed for any of the polymorphisms studied for any of the anatomic subtypes. The limited number of subjects with mutant CYP1A1 alleles did not allow for a calculation of the OR for oral cavity tumors.

To assess dose-response relationship, we have calculated pack-years of smoking (1 pack (20 cigarettes/day) × years of smoking). Smokers were classified as non-to-light/moderate smokers (≤35 pack-years) and heavy smokers (>35 pack-years). Non smokers were analysed together with light/moderate smokers because there were only 2 nonsmokers among the cases and this did not allow for a separate analysis. The odds ratios associated with tobacco consumption by the different genotypes analysed are shown in Table 3. This analysis was not performed for CYP1A1 due to the limited number of patients with mutated alleles and the same happened for alcohol consumption. For the His/Arg genotype of exon 4 mEH, it could be observed that the risk was slightly lower for heavy smokers (OR 0.68; 95% CI 0.25–1.86) than for light smokers (OR 1.08; 95% CI 0.37–3.13) but the associations were not significant for any of the categories of tobacco consumption. The GSTM1 null genotype revealed an inverse pattern, showing an OR of 0.88 (95% CI 0.34–2.34) for light smokers and an OR of 1.40 (95% CI 0.57–3.43) for heavy smokers.

To examine the interaction between these genotypes and the drinking status, we carried out a second stratification analysis. Two categories of alcohol consumption were established. Those that consumed 280 gm/week of alcohol or less (≤2 glasses of wine/beers or 2 liquors a day) were considered as light drinkers, and those that exceeded those values as heavy drinkers. Once again, due to the low number of nondrinkers among the cases (*n* = 2), these subjects were analysed in combination with light drinkers. The risks for the two different categories of alcohol consumption, light and heavy drinkers, are displayed in Table 4. There seems to be a very slight nonsignificant negative association for the His/Arg genotype of exon 4 mEH for both categories of alcohol consumption. The GSTM1 gene has a different effect, with its absence posing a slightly higher association for light drinkers (OR 1.97% CI 0.73–5.35) than for moderate/heavy drinkers (OR 0.69; 95% CI 0.28–1.72).

The interaction between mEH and GSTM1 genes is shown in Table 5. No significant effect was detected for the interaction although the estimated risk was higher for those subjects with the wild-type mEH gene and the null GSTM1 genotype (OR 1.45; 95% CI 0.66–3.17). There was no any apparent effect when both genes were mutated or absent (OR 1.07; 95% CI 0.39–2.92).

## 4. Discussion

Even though previous studies have been undertaken to examine the association between CYP1A1 Ile/Val, mEH, and GSTM1 null polymorphisms, as well as oral and pharyngeal cancers, few investigated the modification of risk associated with tobacco and alcohol consumption, and to our knowledge, this is the first to analyse the gene-gene interactions between these polymorphisms. Our results support the view that there is no significant association for any of these polymorphisms in Caucasians but our data suggest that exon 4 mEH (139 Arg → His) and GSTM1 null polymorphism might modify the risk related to tobacco and alcohol consumption.

We observed that the exon mEH polymorphism 139 Arg → His was mutated in a very similar proportion in cases and controls and the frequencies found (32,3% in controls versus 31,5% in cases) were consistent with the results of the previous investigations that assessed the association between this polymorphism in head and neck tumors in both sexes [32, 37–41]. In these studies, mutated alleles were present in 29.7–39.8% of the control population and in 28.9–39% of the cancer patients. In the present analysis, the estimated risks were not significant for any of the tobacco or alcohol consumption levels but the results were in agreement with those of Wenghoefer et al. [37] that showed that the heterozygous allele (His/Arg) of exon 4 mEH could modulate the risk of head and neck cancer in smokers (OR 0.57; CI 95% 0.34–0.95). In our study, the risk was lower in heavy smokers (OR 0.68; 95% CI 0.25–1.86) than in light smokers (OR 1.08; 95% CI 0.37–3.13) but not significant, maybe influenced by the small sample size.

Enzymaticaly, mEH catalyzes the hydrolysis of arene, alkene, and aliphatic epoxides from polycyclic aromatic hydrocarbons and aromatic amines to trans-dihydrodiols [19]. This reaction is usually regarded as a detoxifying pathway because the majority of metabolites produced are less reactive and can be easily excreted, but, in some instances, these initial trans-dihydrodiol metabolites can be further activated by subsequent P450 catalysis to form highly carcinogenic electrofilic intermediates that can bind covalently to DNA. Such is the case of the 7,8-diol-9,10-epoxide, which is more carcinogenic than the other benzo[a]pyrene diol epoxide formed [42]. Whilst the results of some studies, including the present one, are compatible with the fact that a high or intermediate activity might exert a protective effect in subjects exposed to tobacco products [39, 43, 44], others carried out in various aerodigestive tract cancers find no association or a significantly higher risk for smokers with the exon 4 mEH variant allele [32, 36, 38, 39]; reasons for these inconsistencies are unclear. It could be argued that high activity should be assessed taking into account both the exon 3 and exon 4 mEH polymorphisms. Some authors predicted mEH activity as low, intermediate, or high based on the presence or absence of the two polymorphisms but the results are also contradictory [37, 38, 40, 41, 45, 46]. The study undertaken by Wenghoefer et al. [37] found an association for the single genotypes in head and neck cancers but not the combination genotypes, raising uncertainties in categorizing enzymes. Given the dual role of mEH on the bioactivation/detoxification of carcinogens, it is highly probable that other polymorphisms might influence the formation of carcinogenic metabolites and that gene-gene interactions might exist. In this investigation, we did not find a significant interaction between the GSTM1 and exon 4 mEH polymorphism.

The frequency of the GSTM1 null allele polymorphisms in oral and pharyngeal cancers has been reported to vary greatly depending on the geographical regions [47, 48]. Whilst in Europe and USA the frequencies reported in control populations range from around 49–55.6% [24–32] in Asian and South American countries, this allele is frequently present in less than 49% of the control subjects [49–60]. In the present study, 47.7% of the control population presented the GSTM1 null polymorphism. This is slightly lower than the values found in other Caucasian studies and could be partly due to the fact that only males were included. Our study, like other previous reports on Caucasians [24–26, 28–32], failed to find a significant association between the GSTM1 null polymorphism and oral and pharyngeal cancers. Several studies undertaken in Asian populations showed contradictory results [49, 56–60]. In a very recent meta- and pooled analysis, it was observed that the GSTM1 null polymorphism was significantly associated with risk in Asian and African-American populations in the meta- (OR 1.5; 95% CI 1.3–1.8) and pooled (adjusted OR 2.4, 95% CI 1.1–5.5) analysis, respectively, but not in Caucasians [48]. This discrepancy might be attributed to differences in lifestyle, environmental risk factors, and variations in the activity of other metabolizing enzymes, which often displays genetic polymorphisms that might differ in Caucasians and in other ethnic groups [11, 41, 59, 61].

We did not find a significant interaction between the GSTM1 null polymorphism and tobacco smoking, but we did observe that the risk was slightly higher in heavy smokers than in light/moderate smokers. To verify that these differences were not due to the fact that nonsmokers were analysed together with light/moderate smokers, we carried out a separate analysis leaving out these subjects and found that the variations in the odds ratios were minimum (data not shown). It has been hypothesised that lack of GSTM1 enzyme activity increases cancer susceptibility as a result of a decreased ability to detoxify reactive intermediates of tobacco carcinogens such as benzo[a]pyrene-7,8-diol epoxide, the activated form of benzo[a]pyrene [22] but the results of previous studies undertaken in oral and pharyngeal cancers are inconsistent [26, 54, 56, 57]. Some authors find a lower difference in risk among the genotypes at high dose levels and suggest a dose-response relationship of the enzymatic reaction [48, 57]. In the present study, the relationship between tobacco exposure and these polymorphisms is difficult to assess because there were only 2 nonsmokers among the cases and 6 subjects that smoked for less than 20 years, forcing us to create a category that was light/moderate smokers. Studies with larger number of patients are needed to properly assess this dose response relationship taking into account that ethnic and geographical differences might exist due to the different forms of tobacco consumption and diet intake. In Asian and South American countries, tobacco is usually smoked as “bidis” and the carcinogenic substances found in this form of preparation are different to that in cigarettes, implying that other enzymes different than GSTM1 and CYP1A1 might be involved in the detoxification [28].

Our study suggests that the effect of the GSTM1 null polymorphism is more noticeable among light drinkers, although this association was nonsignificant. This relationship was maintained when we took out the nondrinkers from the analysis (data not shown). This differential effect could be explained by the fact that the GSTM1 isoenzyme, together with the alcohol dehydrogenase, is involved in the oxidation of ethanol to acetaldehyde [30]. Even though the exact mechanisms by which ethanol may exert an influence in oral cancer is still unknown, it has been suggested that acetaldehyde, a known carcinogenic agent, [62] could be responsible for some DNA changes that could lead to cancer [63]. Individuals with a null GSTM1 and high drinkers would not convert ethanol in acetaldehyde and then the absence of the gene would confer them a protective effect for oropharyngeal cancer. Another explanation for these finding could be that ethanol increases the permeability of tobacco carcinogens, such as nitrosonornicotine, across the oral membrane when it is present at low concentrations. At concentrations higher than 50%, no further permeabilization is noted, probably due to the fixative effect of ethanol on the mucosa [64]. It should be highlighted that the alcohol consumption in the Galician population is very high, as other studies have reported [65], and this is a limitation that does not allow for a proper analysis of the interaction.

Even though the CYP1A1 mutation has been shown to increase microsomal activity for converting procarcinogens, including PAH aromatic amines, the results of various reports on smoking-related cancers are inconsistent [11, 32, 47, 66–70]. It has been suggested that the DNA damage may depend on the link of CYP1A1 to other polymorphisms that can affect the CYP1A1 transcription levels, such as polymorphisms for promoter genes, AHR (Ah receptor) genes, or metabolic genes such as GSTM1 [69, 70]. In our study, only 4 of the analysed subjects showed an Ile/Val mutation. Although these frequencies are in accordance with those found by Hahn et al. in a Caucasian population [25], it made it impossible to draw any conclusion for this polymorphism. Confidence intervals obtained were very wide and the distribution of this polymorphism was very similar between cases and controls. In any case, neither the previous meta- and pooled analysis on CYP1A1 and risk of head and neck cancer [47] nor the recently published meta and pooled analysis on oral and pharyngeal cancer found a significant association between CYP1A1 (Ile/Val) polymorphism and oral and pharyngeal cancer [48].

This paper has several limitations. The main one is the small sample size included. This is especially important when analyzing polymorphisms that have a very low frequency in the population, such as CYP1A1 Ile/Val, but also limits the power to identify gene-environmental and gene-gene interactions in any polymorphisms investigated. Nevertheless, it has to be taken into account that for cancers with a low incidence such as oral and pharyngeal ones, 92 cases can be a relatively good number. Another limitation could be the fact that our study was hospital-based and this could result in selection bias. Even though it has been suggested that studies with hospital controls can provide lower risk estimates, since diseases of controls could be associated with the polymorphisms under study, previous meta- and pooled analysis that assessed these polymorphisms on head and neck cancers found no differences for hospital-based studies in relation to population-based studies [47, 66]. The fact that not all the sample was analysed for CYP1A1 polymorphisms limited the power to detect a significant risk.

The present study has also some advantages. One of them is that the participation of cases and controls was very high. All the cases belonged to the same catchment area, which has a unique reference hospital, and were collected consecutively, making them representative of the cases in that area. The controls also belonged to the same area and did not have any symptom or disease related to alcohol or tobacco consumption. Another advantage is the fact that the genes analysed here were of phase I and phase II, which adds value to the results obtained. When studying susceptibility genes in many occasions, both types of genes are not studied and individuals can have susceptibility in type I compensated with the activity of the other type such as it was shown in Park et al. investigation [29].

As a conclusion, it seems that even though none of the three genotypes analysed have a significant association with the risk of oral and pharyngeal cancer in this population, the risks of tobacco and alcohol consumption might be modified by GSTM1 null and exon 4 mEH polymorphisms. The small number of nonsmoking and nondrinking subjects in our population limited our analysis, so we propose that further studies are carried out to clarify this question. Even though we found no significant interaction between the GSTM1null genotype and the exon 4 mEH genotype, the GSTM1 absence did show a slight rise in risk so this should also be investigated taking into account other polymorphisms including the CYP1A1.

## Figures and Tables

**Table 1:** Description of the study individuals.

Variable considered	Cases	Controls
Age (mean, IC95%)	55.0 (52.7–57.3)	59.4 (57.0–61.8)

Cancer location		

Oral	53 (57.6%)	
Pharyngeal	39 (42.4%)	

Cigarette smoking		

Never smoker	2 (2.2%)	49 (37.7%)
Former smoker	17 (18.5%)	42 (32.3%)
Current smoker	73 (79.3%)	39 (30%)

Tobacco consumption (pack-years)		

Percentil 25	33.0	0
Percentil 50	46.5	9.9
Percentil 75	67.5	43.8

Drinking habit		

No alcohol drinking	2 (2.2%)	20 (15.4%)
Light drinker (≤2 glasses of wine/beers or 2 licors a day) (0–280 gm/week)	6 (6.5%)	50 (38.5%)
Moderate drinkers (3–6 drinks/day) (281–840 g/week)	41 (44.6%)	46 (35.4%)
Heavy drinkers (>6 drinks/day) (>840 gm/week)	43 (46.7%)	14 (10.8%)

Alcohol consumption gm/week		

Percentil 25	290	70
Percentil 50	560	145
Percentil 75	840	300

CYP1A1		

Wild type (Ile/Ile)	64 (97%)	90 (97.8%)
Mutation (Ile/Val, Val/Val)	2 (3%)	2 (2.2%)

mEH (139 Arg → His)		

Arg/Arg	63 (68.5%)	88 (67.7%)
Arg/His	25 (27.2%)	41 (31.5%)
His/His	4 (4.3%)	1 (0.8%)

GSTM1		

Present (GSTM1 +)	45 (48.9%)	68 (52.3%)
Absent (GSTM1 −)	47 (51.1%)	62 (47.7%)

Total	92	130

**Table 2:** Risks for oral and pharyngeal cancers associated with polymorphisms in genes CYP1A1, mEH, and GSTM1.

Gene	Cases	Controls	OR crude (CI 95%)*	OR adjusted (CI 95%)^+^
All cancers				

CYP1A1				

No mutated			1.00	1.00
Mutated			0.72 (0.10–5.28)	1.68 (0.18–15.70)

mEH (139His → Arg)				

His/His			1.00	1.00
His/Arg			0.95 (0.52–1.75)	0.81 (0.38–1.71)
Arg/arg			4.30 (0.46–40.07)	4.45 (0.39–50.45)

GSTM1				

Present			1.00	1.00
Absent			1.16 (0.73–1.99)	1.25 (0.65–2.40)

Oral cancer				

CYP1A1				

No mutated	53	64 (97%)	1.00	1.00
Mutated	0	2 (3%)	—	—

mEH (139His → Arg)				

His/His	39 (73,6%)	88 (67,7%)	1.00	1.00
His/Arg	12 (22,6%)	41 (31,5%)	0.74 (0.34–1.58)	0.54 (0.21–1.41)
Arg/arg	2 (3,8%)	1 (0,8%)	3.44 (0.29–40.16)	4.32 (0.32–58.63)

GSTM1				

Present	26 (49,1%)	68 (52,3%)	1.00	1.00
Absent	27 (50,9%)	62 (47,7%)	1.14 (0.60–2.19)	1.20 (0.56–2.18)

Pharyngeal cancer				

CYP1A1				

No mutated	37 (94,9%)	64 (97%)	1.00	1.00
Mutated	2 (5,1%)	2 (3%)	1.74 (0.23–12.92)	4.06 (0.43–38.24)

mEH (139His → Arg)				

His/His	24 (61,5%)	88 (67,7%)	1.00	1.00
His/Arg	13 (33,3%)	41 (31,5%)	1.27 (0.58–2.80)	1.07 (0.42–2.71)
Arg/arg	2 (5,2%)	1 (0,8%)	5.58 (0.47–66.10)	5.74 (0.34–97.71)

GSTM1				

Present	19 (48,7%)	68 (52,3%)	1.00	1.00
Absent	20 (51,3%)	62 (47,7%)	1.20 (0.58–2.48)	1.44 (0.61–3.37)

*Adjusted for age. ^+^Adjusted for age, smoking, and alcohol intake.

**Table 3:** Risks for the different polymorphisms broken down by smoking categories.

Genetic polymorphism	Cases	Controls	OR crude	OR adjusted
Light/moderate smokers (≤35 pack-years)				

CYP1A1				

No mutated	28	41	1.00	1.00
Mutated	1	2	0.76 (0.06–8.90)	1.12 (0.09–13.94)

mEH (139His → Arg)				

His/His	20	58	1.00	1,00
His/Arg	8	24	1.06 (0.40–2.78)	1.08 (0.37–3.13)
Arg/arg	1	1	1.91 (0.11–33.10)	2.82 (0.15–52.41)

GSTM1				

Present	17	43	1.00	1.00
Absent	12	40	0.74 (0.31–1.76)	0.88 (0.34–2.34)

Heavy smokers (>35 pack-years)				

CYP1A1				

No mutated	23	62	1.00	1.00
Mutated	0	1	—	—

mEH (139His → Arg)				

His/His	43	30	1.00	1.00
His/Arg	17	17	0.94 (0.39–2.26)	0.68 (0.25–1.86)
Arg/arg	3	0	—	—

GSTM1				

Present	28	25	1.00	1.00
Absent	35	22	1.50 (0.67–1.33)	1.40 (0.57–3.43)

**Table 4:** Risks for the different polymorphisms broken down by alcohol intake.

Genetic polymorphism	Cases	Controls	OR crude	OR adjusted
Light drinkers (≤280 gm alcohol/week)				

CYP1A1				

No mutated	23	43	1.00	1.00
Mutated	0	2	—	—

mEH (139His → Arg)				

His/His	18	64	1.00	1.00
His/Arg	5	31	0.57 (0.19–1.72)	0.71 (0.22–2.24)
Arg/arg	0	1	—	—

GSTM1				

Present	10	52	1.00	1.00
Absent	13	44	1.55 (0.62–3.88)	1.97 (0.73–5.35)

Moderate/heavy drinkers (>280 gm alcohol/week)				

CYP1A1				

No mutated	67	21	1.00	1.00
Mutated	2	0	—	—

mEH (139His → Arg)				

His/His	45	24	1.00	1.00
His/Arg	20	10	1.28 (0.50–3.31)	0.83 (0.30–2.30)
Arg/arg	4	0	—	—

GSTM1				

Present	35	16	1.00	1.00
Absent	34	18	0.79 (0.34–1.86)	0.69 (0.28–1.72)

**Table 5:** Combination of polymorphisms mEH and GSTM1 and risk of oropharyngeal cancer.

Combination of genetic polymorphisms	Cases	Controls	OR crude	OR adjusted
mEH (Arg/Arg)/GSTM1 present	31	48	1.00	1.00
mEH (Arg/Arg)/GSTM1 absent	32	40	1.22 (0.63–2.36)	1.45 (0.66–3.17)
mEH mutated/GSTM1 present	14	20	1.15 (0.50–2.65)	1,19 (0.43–3.27)
mEH mutated/GSTM1 absent	15	22	1.18 (0.52–2.66)	1.07 (0.39–2.92).

## References

[1] Ferlay J, Bray F, Pisani P, Parkin DM (2004). *GLOBOCAN 2002: Cancer Incidence, Mortality and Prevalence Worldwide*.

[2] Blot WJ, McLaughlin JK, Winn DM (1988). Smoking and drinking in relation to oral and pharyngeal cancer. *Cancer Research*.

[3] Castellsagué X, Quintana MJ, Martínez MC (2004). The role of type of tobacco and type of alcoholic beverage in oral carcinogenesis. *International Journal of Cancer*.

[4] Franceschi S, Talamini R, Barra S (1990). Smoking and drinking in relation to cancers of the oral cavity, pharynx, larynx, and esophagus in Northern Italy. *Cancer Research*.

[5] Lissowska J, Pilarska A, Pilarski P (2003). Smoking, alcohol, diet, dentition and sexual practices in the epidemiology of oral cancer in Poland. *European Journal of Cancer Prevention*.

[6] Moreno-López LA, Esparza-Gómez GC, González-Navarro A, Cerero-Lapiedra R, González-Hernández MJ, Domínguez-Rojas V (2000). Risk of oral cancer associated with tobacco smoking, alcohol consumption and oral hygiene: a case-control study in Madrid, Spain. *Oral Oncology*.

[7] Rosenquist K (2005). Risk factors in oral and oropharyngeal squamous cell carcinoma: a population-based case-control study in southern Sweden. *Swedish Sental Journal. Supplement*.

[8] Bundgaard T, Wildt J, Frydenberg M, Elbrond O, Nielsen JE (1995). Case-control study of squamous cell cancer of the oral cavity in Denmark. *Cancer Causes and Control*.

[9] La Vecchia C, Franceschi S, Favero A, Talamini R, Negri E (1999). Alcohol intake and cancer of the upper digestive tract. Pattern of risk in Italy is different from that in Denmark. *British Medical Journal*.

[10] Grønbæk M, Becker U, Johansen D, Tønnesen H, Jensen G, Sørensen TIA (1998). Population based cohort study of the association between alcohol intake and cancer of the upper digestive tract. *British Medical Journal*.

[11] Bartsch H, Nair U, Risch A, Rojas M, Wikman H, Alexandrov K (2000). Genetic polymorphism of CYP genes, alone or in combination, as a risk modifier of tobacco-related cancers. *Cancer Epidemiology Biomarkers & Prevention*.

[12] Romkes M, White C, Johnson J, Eibling D, Landreneau R, Branch R Expression of cytochrome P450 mRNA in human lung, head and neck tumors and normal adjacent tissues.

[13] Shimada T, Martin MV, Pruess-Schwartz D, Marnett LJ, Guengerich FP (1989). Roles of individual human cytochrome P-450 enzymes in the bioactivation of benzo(*a*)pyrene, 7,8-dihydroxy-7,8-dihydrobenzo(*a*)pyrene, and other dihydrodiol derivatives of polycyclic aromatic hydrocarbons. *Cancer Research*.

[14] Quan T, Reiners JJ, Bell AO, Hong N, States JC (1994). Cytotoxicity and genotoxicity of (±)-benzo[*a*]pyrene-*trans*-7,8-dihydrodiol in CYP1A1-expressing human fibroblasts quantitatively correlate with CYP1A1 expression level. *Carcinogenesis*.

[15] Kawakiri K, Vineis P, Malats N, Lang M (1999). CYP1A1. *Metabolic Polymorphisms and Susceptibility to Cancer*.

[16] Hayashi S, Watanabe J, Nakachi K, Kawajiri K (1991). Genetic linkage of lung cancer-associated MspI polymorphisms with amino acid replacement in the heme binding region of the human cytochrome P450IA1 gene. *The Journal of Biochemistry*.

[17] Guengerich FP (1982). Epoxide hydrolase: properties and metabolic roles. *Reviews in Biochemical Toxicology*.

[18] Beetham JK, Grant D, Arand M (1995). Gene evolution of epoxide hydrolases and recommended nomenclature. *DNA and Cell Biology*.

[19] Fretland AJ, Omiecinski CJ (2000). Epoxide hydrolases: biochemistry and molecular biology. *Chemico-Biological Interactions*.

[20] Hassett C, Robinson KB, Beck NB, Omiecinski CJ (1994). The human microsomal epoxide hydrolase gene (EPHX1): complete nucleotide sequence and structural characterization. *Genomics*.

[21] Hassett C, Aicher L, Sidhu JS, Omiecinski CJ (1994). Human microsomal epoxide hydrolase: genetic polymorphism and functional expression in vitro of amino acid variants. *Human Molecular Genetics*.

[22] Hayes JD, Pulford DJ (1995). The glutathione S-transferase supergene family: regulation of GST and the contribution of the isoenzymes to cancer chemoprotection and drug resistance. *Critical Reviews in Biochemistry and Molecular Biology*.

[23] Coles B, Ketterer B (1990). The role of glutathione and glutathione transferases in chemical cardnogenesis. *Critical Reviews in Biochemistry and Molecular Biology*.

[24] Gronau S, Koenig-Greger D, Jerg M, Riechelmann H (2003). GSTM1 enzyme concentration and enzyme activity in correlation to the genotype of detoxification enzymes in squamous cell carcinoma of the oral cavity. *Oral Diseases*.

[25] Hahn M, Hagedorn G, Kuhlisch E, Schackert HK, Eckelt U (2002). Genetic polymorphisms of drug-metabolizing enzymes and susceptibility to oral cavity cancer. *Oral Oncology*.

[26] Jourenkova-Mironova N, Voho A, Bouchardy C (1999). Glutathione *S*-transferase *GSTM1, GSTM3, GSTP1* and *GSTT1* genotypes and the risk of smoking-related oral and pharyngeal cancers. *International Journal of Cancer*.

[27] Nazar-Stewart V, Vaughan TL, Burt RD, Chen C, Berwick M, Swanson GM (1999). Glutathione *S*-transferase M1 and susceptibility to nasopharyngeal carcinoma. *Cancer Epidemiology Biomarkers & Prevention*.

[28] Matthias C, Bockmühl U, Jahnke V (1998). Polymorphism in cytochrome P450 CYP2D6, CYP1A1, CYP2E1 and glutathione S-transferase, GSTM1, GSTM3, GSTT1 and susceptibility to tobacco-related cancers: studies in upper aerodigestive tract cancers. *Pharmacogenetics*.

[29] Park JY, Muscat JE, Ren Q (1997). *CYP1A1* and *GSTM1* polymorphisms and oral cancer risk. *Cancer Epidemiology Biomarkers & Prevention*.

[30] Coutelle C, Ward PJ, Fleury B (1997). Laryngeal and oropharyngeal cancer, and alcohol dehydrogenase 3 and glutathione S-transferase M1 polymorphisms. *Human Genetics*.

[31] Deakin M, Elder J, Hendrickse C (1996). Glutathione S-transferase GSTT1 genotypes and susceptibility to cancer: studies of interactions with GSTM1 in lung, oral, gastric and colorectal cancers. *Carcinogenesis*.

[32] Boccia S, Cadoni G, Sayed-Tabatabaei FA (2008). *CYP1A1, CYP2E1, GSTM1, GSTT1, EPHX1* exons 3 and 4, and *NAT2* polymorphisms, smoking, consumption of alcohol and fruit and vegetables and risk of head and neck cancer. *Journal of Cancer Research and Clinical Oncology*.

[33] Suzen HS, Guvenc G, Turanli M, Comert E, Duydu Y, Elhan A (2007). The role of GSTM1 and GSTT1 polymorphisms in head and neck cancer risk. *Oncology Research*.

[34] Olshan AF, Weissler MC, Watson MA, Bell DA (2000). *GSTM1, GSTT1, GSTP1, CYP1A1*, and *NAT1* polymorphisms, tobacco use, and the risk of head and neck cancer. *Cancer Epidemiology Biomarkers & Prevention*.

[35] Bell DA, Taylor JA, Paulson DF, Robertson CN, Mohler JL, Lucier GW (1993). Genetic risk and carcinogen exposure: a common inherited defect of the carcinogen-metabolism gene glutathione S-transferase M1 (GSTM1) that increases susceptibility to bladder cancer. *Journal of the National Cancer Institute*.

[36] Zhao H, Spitz MR, Gwyn KM, Wu X (2002). Microsomal epoxide hydrolase polymorphisms and lung cancer risk in non-hispanic whites. *Molecular Carcinogenesis*.

[37] Wenghoefer M, Pesch B, Harth V (2003). Association between head and neck cancer and microsomal epoxide hydrolase genotypes. *Archives of Toxicology*.

[38] Lacko M, Roelofs HMJ, te Morsche RHM (2008). Microsomal epoxide hydrolase genotypes and the risk for head and neck cancer. *Head & Neck*.

[39] To-Figueras J, Gené M, Gómez-Catalán J (2002). Microsomal epoxide hydrolase and glutathione S-transferase polymorphisms in relation to laryngeal carcinoma risk. *Cancer Letters*.

[40] Jourenkova-Mironova N, Mitrunen K, Bouchardy C, Dayer P, Benhamou S, Hirvonen A (2000). High-activity microsomal epoxide hydrolase genotypes and the risk of oral, pharynx, and larynx cancers. *Cancer Research*.

[41] Park JY, Schantz SP, Lazarus P (2003). Epoxide hydrolase genotype and orolaryngeal cancer risk: interaction with GSTM1 genotype. *Oral Oncology*.

[42] Shou M, Gonzalez FJ, Gelboin HV (1996). Stereoselective epoxidation and hydration at the K-region of polycyclic aromatic hydrocarbons by cDNA-expressed cytochromes P450 1A1, 1A2, and epoxide hydrolase. *Biochemistry*.

[43] Heckbert SR, Weiss NS, Hornung SK, Eaton DL, Motulsky AG (1992). Glutathione S-transferase and epoxide hydrolase activity in human leukocytes in relation to risk of lung cancer and other smoking-related cancers. *Journal of the National Cancer Institute*.

[44] Amador AG, Righi PD, Radpour S (2002). Polymorphisms of xenobiotic metabolizing genes in oropharyngeal carcinoma. *Oral Surgery, Oral Medicine, Oral Pathology, Oral Radiology, & Endodontics*.

[45] Kiyohara C, Yoshimasu K, Takayama K, Nakanishi Y (2006). EPHX1 polymorphisms and the risk of lung cancer: a HuGE review. *Epidemiology*.

[46] Lee WJ, Brennan P, Boffetta P (2002). Microsomal epoxide hydrolase polymorphisms and lung cancer risk: a quantitative review. *Biomarkers*.

[47] Hashibe M, Brennan P, Strange RC (2003). Meta- and pooled analysis of *GSTM1, GSTT1, GSTP1*, and *CYP1A1* genotypes and risk of head and neck cancer. *Cancer Epidemiology Biomarkers & Prevention*.

[48] Varela-Lema L, Taioli E, Ruano-Ravina A (2008). Meta-analysis and pooled analysis of GSTM1 and CYP1A1 polymorphisms and oral and pharyngeal cancers: a HuGE-GSEC review. *Genetics in Medicine*.

[49] Soya SS, Vinod T, Reddy KS, Gopalakrishnan S, Adithan C (2007). Genetic polymorphisms of glutathione-*S*-transferase genes (*GSTM1, GSTT1* and *GSTP1*) and upper aerodigestive tract cancer risk among smokers, tobacco chewers and alcoholics in an Indian population. *European Journal of Cancer*.

[50] Sikdar N, Paul RR, Roy B (2004). Glutathione *S*-transferase *M3 (A/A)* genotype as a risk factor for oral cancer and leukoplakia among Indian tobacco smokers. *International Journal of Cancer*.

[51] Buch SC, Notani PN, Bhisey RA (2002). Polymorphism at *GSTM1, GSTM3* and *GSTT1* gene loci and susceptibility to oral cancer in an Indian population. *Carcinogenesis*.

[52] Sreelekha TT, Ramadas K, Pandey M, Thomas G, Nalinakumari KR, Pillai MR (2001). Genetic polymorphism of *CYP1A1, GSTM1* and *GSTT1* genes in Indian oral cancer. *Oral Oncology*.

[53] Gattá GJF, de Carvalho MB, Siraque MS (2006). Genetic polymorphisms of *CYP1A1, CYP2E1, GSTM1*, and *GSTT1* associated with head and neck cancer. *Head & Neck*.

[54] Xie H, Hou L, Shields PG (2003). Metabolic polymoryphisms, smoking, and oral cancer in Puerto Rico. *Oncology Research*.

[55] Drummond SN, De Marco L, Noronha JCM, Gomez RS (2004). GSTM1 polymorphism and oral squamous cell carcinoma. *Oral Oncology*.

[56] Kietthubthew S, Sriplung H, Au WW (2001). Genetic and environmental interactions on oral cancer in Southern Thailand. *Environmental and Molecular Mutagenesis*.

[57] Sato M, Sato T, Izumo T, Amagasa T (1999). Genetic polymorphism of drug-metabolizing enzymes and susceptibility to oral cancer. *Carcinogenesis*.

[58] Sato M, Sato T, Izumo T, Amagasa T (2000). Genetically high susceptibility to oral squamous cell carcinoma in terms of combined genotyping of *CYP1A1* and *GSTM1* genes. *Oral Oncology*.

[59] Nomura T, Noma H, Shibahara T, Yokoyama A, Muramatusu T, Ohmori T (2000). Aldehyde dehydrogenase 2 and glutathione *S*-transferase M1 polymorphisms in relation to the risk for oral cancer in Japanese drinkers. *Oral Oncology*.

[60] Kihara M, Kihara M, Kubota A, Furukawa M, Kimura H (1997). *GSTM1* gene polymorphism as a possible marker for susceptibility to head and neck cancers among Japanese smokers. *Cancer Letters*.

[61] Marques CFS, Koifman S, Koifman RJ, Boffetta P, Brennan P, Hatagima A (2006). Influence of *CYP1A1, CYP2E1, GSTM3* and *NAT2* genetic polymorphisms in oral cancer susceptibility: results from a case-control study in Rio de Janeiro. *Oral Oncology*.

[62] IARC (International Agency for Research on Cancer) (1998). *Alcohol Drinking*.

[63] Ristow H, Obe G (1978). Acetaldehyde induces cross-links in DNA and causes sister-chromatid exchanges in human cells. *Mutation Research*.

[64] Howie NM, Trigkas TK, Cruchley AT, Wertz PW, Squier CA, Williams DM (2001). Short-term exposure to alcohol increases the permeability of human oral mucosa. *Oral Diseases*.

[65] Ruano-Ravina A, Figueiras A, Barros-Dios JM (2004). Type of wine and risk of lung cancer: a case-control study in Spain. *Thorax*.

[66] Hung RJ, Boffetta P, Brockmöller J (2003). CYP1A1 and GSTM1 genetic polymorphisms and lung cancer risk in Caucasian non-smokers: a pooled analysis. *Carcinogenesis*.

[67] Taioli E, Gaspari L, Benhamou S (2003). Polymorphisms in CYP1A1, GSTM1, GSTT1 and lung cancer below the age of 45 years. *International Journal of Epidemiology*.

[68] Vineis P, Veglia F, Anttila S (2004). CYP1A1, GSTM1 and GSTT1 polymorphisms and lung cancer: a pooled analysis of gene-gene interactions. *Biomarkers*.

[69] Crofts F, Taioli E, Trachman J (1994). Functional significance of different human CYPlAl genotypes. *Carcinogenesis*.

[70] Rojas M, Alexandrov K, Cascorbi I (1998). High benzo[a]pyrene diol-epoxide DNA adduct levels in lung and blood cells from individuals with combined CYP1A1 MspI/MspI-GSTM1*0/*0 genotypes. *Pharmacogenetics*.

